# A Prospective Evaluation of Intraoperative Indocyanine Green Fluorescence Angiography for Soft Tissue Sarcomas

**DOI:** 10.5435/JAAOSGlobal-D-21-00187

**Published:** 2021-08-19

**Authors:** Benjamin K. Wilke, Douglas S. Schultz, Maria T. Huayllani, Daniel Boczar, Aaron C. Spaulding, Courtney Sherman, Peter Murray, Antonio J. Forte

**Affiliations:** From the Department of Orthopedic Surgery (Dr. Wilke, Dr. Schultz, Dr. Sherman, Dr. Murray, Dr. Forte), the Division of Plastic Surgery (Dr. Huayllani, Dr. Boczar, Dr. Forte); and the Robert D. and Patricia E. Kern Center for the Science of Health Care Delivery (Dr. Huayllani, Dr. Boczar, Dr. Spaulding, Dr. Forte), Mayo Clinic, Jacksonville, Florida.

## Abstract

**Introduction:**

Postoperative wound complications after resection of soft-tissue sarcomas are challenging. Indocyanine green (ICG) angiography has previously been used to predict wound complications, but not for soft-tissue sarcomas. We aimed to evaluate whether this technology could help lower wound complications after soft-tissue sarcoma resections.

**Materials and Methods:**

We conducted a prospective study from 10/2017 to 9/2019 using ICG angiography during sarcoma resection surgery. Rates of wound complications were compared with a historical control consisting of surgeries before utilization of ICG angiography.

**Results:**

A total of 88 patients were included in the study. We found significantly lower rates of infection (11.8% versus 38%; *P* = 0.03) and wound dehiscence (11.8% versus 42.3%; *P* = 0.02) in the ICG angiography cohort compared with the historical controls.

**Conclusion:**

ICG angiography use during soft-tissue sarcoma resections is promising technology and warrants further investigation to help reduce postoperative complications.

Postoperative wound complications after resection of a soft-tissue sarcoma are a challenging issue. Historically, rates of wound complications after preoperative radiation therapy and surgical resection have been reported to be greater than 30%.^1-3^ Unfortunately, little progress has been made over time, with similar rates reported in more recent literature.^4,5^

Wound dehiscence and infection are the predominant causes of postoperative wound complications in these patients. Previous studies have reported on risk factors for developing these complications and include preoperative radiation, size of the tumor, location of the tumor, smoking status, diabetes, and vascular disease.^3,4,5,6,7^ A number of interventions such as more aggressive flap coverage, hyperbaric oxygen therapy, silver dressings, or wound vacuum technology have achieved some success in mitigating the wound complication risk in this population.^7-9^ However, there is still ample room to improve complication rates.

A previous limitation for surgeons treating sarcomas was the inability to predict the tissue's capacity to heal in real time, during the operative setting. Knowing the tissue at risk would allow the surgeon to change the reconstruction plan at the time of surgery to decrease the risk of developing postoperative wound complications. Indocyanine green (ICG) angiography provides the surgeon this ability and has successfully been used in plastic surgery, general surgery, and more recently in orthopaedic surgery, to predict the survival of tissue and planned flaps. ^10,11,12,13^ We recently reported on ICG angiography use in sarcoma surgery, demonstrating that it was predictive of developing a postoperative wound complication, especially in the lower extremity.^14^ What is unknown, however, is whether use of this technology will translate to lower postoperative wound complication rates.

We hypothesized that ICG angiography use would reduce postoperative wound complications, specifically wound dehiscence and infection, in soft-tissue sarcoma resections after preoperative radiation therapy. We conducted a prospective study using intraoperative ICG angiography and compared the wound complication rates with historical controls.

## Methods

After institutional review board approval, a prospective study was conducted from 10/2017 to 9/2019 to evaluate the ability of intraoperative indocyanine green fluorescence angiography to reduce postoperative wound complications after soft-tissue sarcoma resection. Eligible patients included those with a diagnosis of a nonmetastatic soft-tissue sarcoma who were scheduled for elective surgical resection. Patients were then included in the study if ICG angiography was used to evaluate and manage their reconstruction. Exclusion criteria included patients with an iodine allergy who could not undergo ICG angiography due to cross-reactivity.

During the surgical procedure, the sarcoma resection was done routinely. The reconstruction was then done with the assistance of the microvascular team if a local flap or free flap was required. After wound closure, and before application of the dressing, the incision was evaluated using ICG fluorescence angiography. One vial of ICG powder (25 mg/vial) was reconstituted with 10 mL of sterile water to create a 2.5 mg/mL solution. Five milliliters of the solution was injected intravenously, followed by a 10-mL sterile saline flush. Images were then captured using the PDE-NEO II handheld device (Mitaka). Areas of hypoperfusion were marked with a surgical pen. These areas were then excised until the entire incision appeared well perfused, as evaluated with follow-up fluorescence angiography. Rates of wound dehiscence and infection were recorded. Dehiscence was defined as an unexpected separation of the tissue layers, whereas infection was defined as any patient requiring antibiotic therapy secondary to wound drainage or cellulitis. Patients were followed postoperatively until the incision had healed, and then every 4 months for surveillance imaging, per our institutional guidelines.

The prospective intraoperative angiography cohort was then compared with a cohort (control group) consisting of patients who underwent soft-tissue sarcoma resection before the institutional adoption of this technology for sarcoma patients. Chart review was done on these historical cohort patients, who underwent surgery from 1/2014 to 5/2017, to obtain patient demographics and surgical details.

### Statistical Analysis

Patient demographics, tumor characteristics, and rates of complications were described and compared using the Fisher exact test for categorical variables. The Student *t*-test was used to compare means of continuous variables. A *P* value of less than 0.05 was considered significant. Statistical Package for the Social Sciences (SPSS), version 25, software (SPSS) was used for the analyses.

## Results

Eighty-eight patients were included in the study. Seventeen patients (19%) underwent sarcoma resection with wound closure guided by intraoperative fluorescence angiography, whereas 71 patients (81%) served as historical controls.

In the intraoperative fluorescence angiography cohort, there were nine males (52.9%) and eight females (47.1%). The average age at the time of surgery was 58 years (range 29 to 86 years), whereas the average body mass index was 27 kg/m^2^ (range 20 to 36.8 kg/m^2^). Patient demographics compared with historical controls are listed in Table 1, with the only significant difference being a higher rate of peripheral vascular disease documented in the fluorescence angiography cohort.

**Table 1:** Patient Demographics Compared With Historical Controls

Variables	Control		Fluorescence Angiography		*P*
	N = 71	81.0%	N = 17	19.0%	
Age (mean ± SD)	59.6	17.5	57.7	16.9	0.69
Sex					>0.99
Males	37	52.1%	9	52.9%	
Females	34	47.9%	8	47.1%	
BMI, kg/m^2^ (mean ± SD)	29.53	7.4	27.2	5.1	0.23
Smoking					0.62
No	39	54.9%	12	70.6%	
Yes	9	12.7%	1	5.9%	
Only history	23	32.4%	4	23.5%	
Steroids use					0.59
No	65	91.5%	16	94.1%	
Yes	6	8.5%	1	5.9%	
Diabetes					0.38
No	62	87.3%	16	94.1%	
Yes	9	12.7%	1	5.9%	
PVD					**0.04**
No	69	97.2%	14	82.4%	
Yes	2	2.8%	3	17.6%	
CAD					0.42
No	58	81.7%	13	76.5%	
Yes	13	18.3%	4	23.5%	

Bold = P < 0.05. BMI = body mass index, CAD = coronary artery disease, PVD = peripheral vascular disease

In the fluorescence angiography cohort, 15 patients (88.2%) underwent radiation, all of which was given preoperatively (neoadjuvant) per our institutional protocol. The average radiation dose was 50 Gy. In addition, five patients (29.4%) received chemotherapy; two received neoadjuvant therapy (11.8%), whereas three (17.6%) received adjuvant treatment. We found no differences in the rates of radiation or chemotherapy given between the angiography cohort and the historical controls (*P* > 0.05) (Table 2).

**Table 2:** Tumor Characteristics and Postoperative Complications

Variables	Control		Fluorescence Angiography		*P*
	N = 71	81.0%	N = 17	19.0%	
Tumor location					0.44
Upper extremity	14	19.7%	5	29.4%	
Lower extremity	45	63.4%	12	70.6%	
Thorax	6	8.5%	0	0.0%	
Pelvis/genitalia	6	8.5%	0	0.0%	
Tumor size (cm)					
Mean ± SD	8.86	7.6	7.88	5.5	0.61
Radiation therapy					0.07
No	26	36.6%	2	11.8%	
Yes	45	63.4%	15	88.2%	
Chemotherapy					0.62
No	50	70.4%	12	70.6%	
Yes	21	29.6%	5	29.4%	
Closure					0.17
Primary	33	46.5%	7	41.2%	
Local flap	26	36.6%	4	23.5%	
Free flap	7	9.9%	2	11.8%	
Skin graft	4	5.6%	3	17.6%	
Free flap and skin graft	1	1.4%	0	0.0%	
Local flap and skin graft	0	0.0%	1	5.9%	
Infection					**0.03**
No	44	62.0%	15	88.2%	
Yes	27	38.0%	2	11.8%	
Wound dehiscence					**0.02**
No	41	57.7%	15	88.2%	
Yes	30	42.3%	2	11.8%	

Bold = P < 0.05.

The average size of the tumors was 7.88 cm in the angiography cohort (range: 1 to 22 cm). Five tumors (29.4%) were located in the upper extremity, and 12 (70.6%) were found in the lower extremity. Six tumors (35%) were superficial to the fascia, and 11 tumors (65%) were deep. All 17 patients underwent resection with a wide margin. We found no differences in tumor characteristics between the prospective cohort and the historical controls (*P* > 0.05).

In the angiography cohort, seven wounds (41.2%) underwent primary closure. Four (23.5%) were closed with local flaps, whereas two (11.8%) required a free flap. Three patients (17.6%) underwent a split-thickness skin graft. Thirteen patients (77%) had an incisional wound vac placed at the time of wound closure. We found no differences in the types of closure between the cohorts (*P* > 0.05).

**Figure 1:**
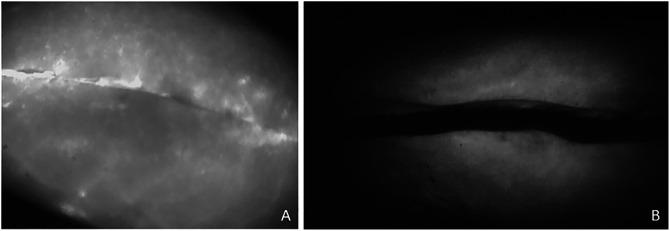
Intraoperative angiography images demonstrating adequate perfusion (**A**) and hypoperfusion (**B**) along the incisions.

Intraoperative fluorescence angiography demonstrated that five patients (29.4%) had hypovascular areas of skin along their incision after wound closure (Figure 1). All five of these patients had this skin excised during the procedure. Two of these patients (11.8%) developed a wound complication postoperatively; both complications were due to wound dehiscence. The remaining three patients (17.6%) did not develop a wound complication.

There were an additional three patients (17.6%) who developed wound complications that were not predicted by intraoperative fluorescence angiography. All three of these patients had issues related to the partial breakdown of a split-thickness skin graft. Two of the patients (11.8%) developed a superficial infection; one required a return to the operating room for a débridement.

When comparing rates of postoperative infections in the fluorescence angiography group with our historical controls, we found a statistically significant lower rate of infections in the angiography cohort (11.8% versus 38%; *P* = 0.03). Similarly, rates of wound dehiscence were also significantly lower in the angiography cohort compared with the control group (11.8% versus 42.3%; *P* = 0.02).

## Discussion

Wound complications after sarcoma resection are a challenging issue. Preoperative radiation is associated with a higher rate of developing these complications than with postoperative treatment. This was demonstrated by O'Sullivan et al^5^, who reported a 35% wound complication rate in their preoperative cohort, compared with 17% when patients received radiation therapy in the postoperative setting. Despite this, many clinicians, including our center, still choose preoperative radiation due to other advantages such as a smaller radiation field, lower radiation dose, and less long-term fibrosis.^15^

Clinicians have attempted to reduce the wound complication rate with preoperative radiation therapy to more similarly match the risk observed with postoperative treatment by identifying risk factors for developing complications as well as using techniques, such as hyperbaric oxygen or wound vacuum technology, to reduce the risk. So far, however, we have not seen a substantial decrease in wound complication rates.^4,5^ We think a major limitation has been the inability to directly visualize the vascular supply to the tissue intraoperatively, which would allow removal of tissue with poor perfusion and healing potential at the time of the index surgery.

ICG angiography is a technology that allows direct visualization of tissue perfusion intraoperatively. It distributes entirely in the intravascular space, allowing accurate visualization of tissue perfusion. In addition, it has a very short half-life (3 minutes), which is ideal if recurrent dosing is required.^16,17^ It has been shown to accurately detect perfusion abnormalities and reduce wound healing complications in a variety of procedures.^13,17,18,19,20^ For example, in a study by Rinker,^10^ skin-sparing mastectomies demonstrated decreased skin flap necrosis when evaluated intraoperatively with ICG compared with direct visualization of skin perfusion.

Before our institutional adoption of ICG angiography for soft-tissue sarcoma resections, we had an infection rate of 38% and a wound dehiscence rate of 42.3%, similar to complication rates reported in the literature associated with preoperative radiation.^1-3,5^ After including this technology in our surgical reconstruction plan, we have demonstrated a significant decrease in wound dehiscence rates and infections, more closely approximating complication rates observed with postoperative radiation therapy at 11.8% in our cohort.

There were three patients in our prospective cohort who developed partial breakdown of a split-thickness skin graft. None of these patients had hypoperfusion detected with the ICG angiography. Given that split-thickness skin grafts require more than just a well-vascularized tissue bed to heal, we do not think that ICG angiography is helpful in predicting success for split-thickness skin grafts.

There are several limitations to this study. Namely, we have a small sample size. Despite this, we were able to demonstrate a statistically significant difference in wound dehiscence and infection rates between the cohorts. In addition, the comparison group was retrospective in nature. A prospective randomized trial would be a higher level of evidence. Last, this study was conducted at a single institution. Larger, multi-institutional studies are needed to verify these results. Despite these limitations, we think that this study is important because it has shown a drastically reduced rate of wound complications when using intraoperative indocyanine angiography during sarcoma resection.

## Conclusion

In conclusion, we found statistically significantly lower wound complication rates, specifically for infection and wound dehiscence, after sarcoma resections when using intraoperative ICG angiography. We recommend continued use of this technology.
